# Strategies for the Voltammetric Detection of Loop-Mediated Isothermal Amplification

**DOI:** 10.3390/mi14020472

**Published:** 2023-02-18

**Authors:** Jesse M. Marangoni, Kenneth K. S. Ng, Arezoo Emadi

**Affiliations:** 1 Department of Chemistry and Biochemistry, University of Windsor, Windsor, ON N9B 3P4, Canada; 2 Department of Electrical and Computer Engineering, University of Windsor, Windsor, ON N9B 3P4, Canada

**Keywords:** electrochemical biosensors, voltammetry, loop-mediated isothermal amplification

## Abstract

Loop-mediated isothermal amplification (LAMP) is rapidly developing into an important tool for the point-of-use detection of pathogens for both clinical and environmental samples, largely due to its sensitivity, rapidity, and adaptability to portable devices. Many methods are used to monitor LAMP, but not all are amenable to point-of-use applications. Common methods such as fluorescence often require bulky equipment, whereas colorimetric and turbidimetric methods can lack sensitivity. Electrochemical biosensors are becoming increasingly important for these applications due to their potential for low cost, high sensitivity, and capacity for miniaturization into integrated devices. This review provides an overview of the use of voltammetric sensors for monitoring LAMP, with a specific focus on how electroactive species are used to interface between the biochemical products of the LAMP reaction and the voltammetric sensor. Various strategies for the voltammetric detection of DNA amplicons as well as pyrophosphate and protons released during LAMP are presented, ranging from direct DNA binding by electroactive species to the creative use of pyrophosphate-detecting aptamers and pH-sensitive oligonucleotide structures. Hurdles for adapting these devices to point-of-use applications are also discussed.

## 1. Introduction

The detection of pathogens in clinical or environmental samples is a critical component in the management of disease, both at the individual and public health levels. The ideal pathogen detection assay would be sensitive, specific, rapid, simple, and inexpensive, but there exists a technological gap between tests with high sensitivity/specificity and those with high rapidity/simplicity [1]. At one extreme, nucleic acid detection by polymerase-chain reaction (PCR) is both highly specific and highly sensitive, but its requirements for specialized equipment and operators, and a relatively long sample processing and amplification time, often preclude its use at point-of-care [2,3]. At the other extreme, lateral-flow assays (LFA), which typically detect pathogens immunologically, are rapid to use and do not require special training, but they have comparatively low sensitivity and consequently a higher potential for false-negative results [1].

As a result, there is currently an open niche for devices which combine the rapidity and ease-of-use of LFA with the sensitivity and specificity of PCR. To this end, much work has been done on improving the sensitivity of immunoassays. For example, LFA sensitivity can be increased through enzymatic signal amplification [4] or by using fluorescent probes [5]. However, the modifications required to approach PCR-level sensitivity with these enhanced LFAs also increase the complexity of the assay and introduce additional requirements for reading equipment, which abrogate the LFA advantages of simplicity and rapidity [1]. Immunological-based sensors outside of the LFA format have also been developed. For example, antibody-functionalized quartz crystal microbalances (QCM) have been successfully used to detect pathogens with improved sensitivity over traditional LFAs, and further signal amplification can be achieved by implementing a sandwich method with antibody-conjugated gold nanoparticles [6]. As with the enhanced LFAs, however, increasing sensitivity also increases assay complexity and processing time.

In contrast to immunoassays, nucleic acid detection has several distinct advantages, including the high specificity of recognition, the relative ease of developing new oligonucleotide primers for new assays and/or novel pathogens, and the inherent potential for exponential amplification arising from the nature of the replicative mechanism. PCR is the gold-standard for nucleic acid amplification, and great progress has been made in adapting it for point-of-use applications [7], including with electrochemical sensors [8]. Despite these advances, however, PCR still has a relatively long time-to-detection, and the requirement for thermocycling increases device complexity. As a possible solution to these shortcomings, isothermal amplification techniques are becoming increasingly popular in the development of point-of-use devices [9,10,11]. Of the many isothermal amplification techniques thus far developed, loop-mediated isothermal amplification (LAMP) has become popular due to its rapid, high-efficiency amplification of very low copy numbers of the template, and the theoretical potential for very high specificity [12]. For example, LAMP has been used to create simple point-of-care tests for HIV [13] and SARS-CoV2 [14].

Myriad detection methods have been used to monitor LAMP, and they have been extensively reviewed [15,16]. Presently, the most common detection strategy is optical, and, because of the extremely high efficiency amplification, detection can often be by naked-eye colorimetric (e.g., color change of a pH indicator) or turbidimetric (precipitation of magnesium pyrophosphate) observations. Due to the lack of a requirement for detection equipment, this is very attractive for point-of-use applications [17]. However, these relatively simple methods typically lack sensitivity and the capacity for accurate quantification. Fluorescence-based LAMP assays have also become popular due to their high sensitivity that rivals and often even surpasses PCR [18]. Although progress is being made in integrating the optical detection of LAMP for point-of-use [14], the general requirement for bulky and expensive detection equipment remains an obstacle.

As an alternative to optical assays, LAMP has been combined with a wide variety of electronic transducers. For example, a combination LAMP/QCM device has shown similar sensitivity to PCR [19], ion-sensitive field-effect transistors (ISFET) have been employed to monitor LAMP reactions by detecting changes in pH due to the release of hydrogen ions during DNA synthesis [20], and there are numerous examples of electrochemical sensors being used [10]. Electrochemical detection is advantageous for its relatively low cost and adaptability to integrated, miniaturized devices [21]. In particular, voltammetric biosensors have been thoroughly developed due to their potential for high-sensitivity detection. Voltammetry monitors the faradaic current generated by the reduction and/or oxidation of an electroactive species at a working electrode as the electric potential is swept against a reference electrode. Because of its relative simplicity, this method has long been exploited in the development of biosensors, but its adaptability has also allowed its use in many novel and creative designs. This review focuses on the interface between LAMP and the voltammetric electrochemical sensor; that is, on the varied strategies implemented to use electroactive molecules to indirectly monitor LAMP.

## 2. Principles of LAMP

LAMP was first developed by Notomi et al. (2000) to address some of the problems of other contemporaneous isothermal amplification methods, namely relatively low specificity due to low stringency primer annealing [22]. Whereas PCR requires thermocycling between three different temperatures for dsDNA denaturation, primer annealing, and DNA extension, LAMP is conducted isothermally at 60–65 °C, which is the temperature optimum for the polymerase that is typically used. Amplification is achieved by using a strand-displacing polymerase, often *Bst*, that allows the polymerase to displace the previously synthesized non-template strand of a DNA duplex without the need for thermal denaturation.

**Figure 1:**
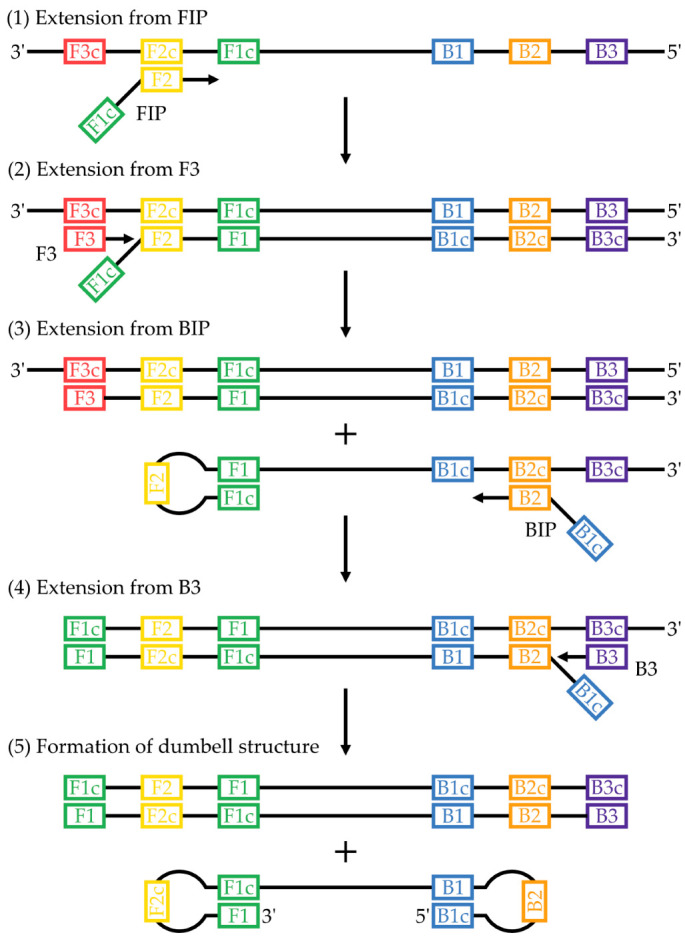
Mechanism of LAMP up to the formation of the dumbbell structure. (1) The F2 region of primer FIP hybridizes to the F2 region of the template, and replication is initiated. This introduces a region of self-complementarity in the newly synthesized strand. (2) Extension from primer F3 displaces the previously synthesized strand. (3) B2 of BIP hybridizes with B2c of the displaced strand and replication occurs. (4) Extension from B3 displaces the previously synthesized strand. (4) The self-complementary regions form a self-priming dumbbell structure that feeds into the cycling and elongation phases of LAMP. Figure is adapted from [22,23], which also provide a detailed explanation of the entire LAMP mechanism.

The multistep mechanism of DNA amplification in LAMP has been described comprehensively in the seminal articles in which it was introduced [22,23]. In brief, primers are designed to recognize a template with six distinct regions. These regions are designated from 5′ to 3′, B3, B2, B1, F1c, F2c, and F3c on one strand and F3, F2, F1, B1c, B2c, and B3c on the complementary strand (F1, for example, is complementary to F1c). In the original method, four primers recognizing these six regions were used: two inner primers, a forward inner primer (FIP) and backward inner primer (BIP), and two outer primers, F3 and B3. FIP contains regions F1c and F2 while BIP contains regions B1c and B2. Although LAMP can be initiated on double-stranded templates through strand-invasion by the primers [24], a single input strand will be considered here for simplicity: 3′-F3c-F2c-F1c-B1-B2-B3-5′. The F2 region of FIP hybridizes with F2c in the template to initiate replication (Figure 1, step 1). Extension from primer F3, hybridized to F3c, by *Bst* displaces the strand previously synthesized from FIP (Figure 1, step 2). Region B2 of BIP can hybridize to B2c of the displaced strand and initiate replication on it (Figure 1, step 3), which is itself displaced by the extension from B3 (Figure 1, step 4). The extension from both FIP and BIP introduces regions of self-complementarity to the newly synthesized strand, which can then self-hybridize to form a characteristic double stem-loop, or dumbbell, structure (Figure 1, step 5). The importance of this structure lies in its ability to self-prime replication [15]. Continued cycling of replication initiation between (1) the 3′ end of the dumbbell, and (2) the inner primers hybridizing to the dumbbell loop, allows for exponential amplification and generates a mixture of stem-loops and what have been described as cauliflower-like structures [22].

Detection strategies for LAMP commonly rely on monitoring the generation of products, including the LAMP amplicons themselves as well as pyrophosphate and protons, both products of the nucleotidyl-transfer reaction which occurs as nucleoside triphosphates are incorporated into growing DNA strands. For the standard optical assays, (1) DNA amplicons can be monitored by DNA-binding fluorescent dyes through fluorescence measurements, (2) pyrophosphate can be monitored by precipitation with magnesium cations (included in the reaction as a cofactor of the polymerase) through turbidimetric measurements, or (3) protons can be monitored by pH-indicating dyes through colorimetry.

In the decades since its introduction, the LAMP method has been further refined to increase its usefulness. Later work added two additional loop primers (six total recognizing eight regions) that greatly improved efficiency, such that amplification and detection could be achieved in less than an hour [23]. The inclusion of a thermostable reverse transcriptase in the reaction mixture makes it easily adaptable to RNA templates and has been used to develop diagnostic assays for SARS-CoV2, for example [25]. Multiplex assays are also possible by utilizing differently labelled oligonucleotide hydrolysis probes [26], and digital format LAMP, in which single copies of target nucleic acid are amplified in picolitre-sized microwells [27] or emulsified droplets [28], allows for absolute quantification of the template. Progress in the development of LAMP has been recently reviewed [15,29,30].

LAMP offers many inherent advantages over other isothermal amplification methods and even PCR. A high degree of amplification can be achieved rapidly, with single copies of target nucleic acids being amplified a billion-fold [12] and threshold times, which is the time for the monitored signal to rise above the baseline, being reported as less than 10 min in SARS-CoV2 clinical samples, for example [31]. The higher temperature used relative to other isothermal methods creates greater stringency for primer annealing, which, combined with the use of 4–6 primers recognizing 6–8 regions, allows, at least theoretically, greater specificity towards a given target sequence [22]. LAMP is more tolerant than PCR to interfering compounds present in clinical or environmental samples, and so prior DNA purification can often be simplified or omitted entirely, which greatly simplifies the procedure for point-of-use applications [32]. LAMP also does not require a denatured template and can be combined with a reverse transcriptase in the same reaction to amplify RNA. All of these attributes combine to create an excellent tool for the rapid and efficient amplification of nucleic acids. It has become by far the most-used isothermal amplification method [12,15], and is already authorized for use in pathogen detection by a number of diverse organizations. For example, the European and Mediterranean Plant Protection Organization (EPPO; Paris, France) has validated it for pest detection, and it meets all of the World Health Organization (WHO; Geneva, Switzerland)’s criteria for diagnostic tests, including sensitivity, specificity, simplicity, and rapidity [33].

## 3. Voltammetric Detection Strategies

The application of voltammetry to monitoring LAMP ranges from simple assays monitoring DNA-binding reporters to complex ones involving, for example, additional enzymes or tertiary nucleic acid structures. An extensive overview of voltammetry is outside the scope of this review, but excellent guides are available such as that by Elgrishi et al. (2018) [34]. In brief, voltammetry measures the oxidation and reduction of chemical species. Whereas in chemical redox reactions, electrons are transferred between two chemical species, in voltammetry, electrons are transferred from an electrode to a chemical species, or vice versa, and such a species is said to be electroactive. The potential at which electron transfers occur is characteristic of the electroactive species itself, and a typical voltammetry experiment will sweep across a range of voltages. When the necessary potential is reached, electron transfer occurs, and this movement of electrons represents a current, called a faradaic current, which can be measured.

An important concept in voltammetry is that, as the electroactive species near to the electrode become reduced or oxidized, the rate-limiting step of further electron transfer reactions becomes the diffusion of the electroactive species from the bulk solution to the electrode surface. Thus, anything that can change the diffusivity of the electroactive species, or that can otherwise concentrate it at or away from the electrode surface, will change the resulting faradaic current. The general strategy for the voltammetric detection of LAMP is therefore to use the production of LAMP products (DNA amplicons, pyrophosphate, and protons) to influence the diffusivity of an electroactive species and/or to alter its effective concentration at the electrode.

### 3.1. Monitoring DNA Polymerization

#### 3.1.1. Sequestration of Electroactive Species from the Electrode

**Figure 2:**
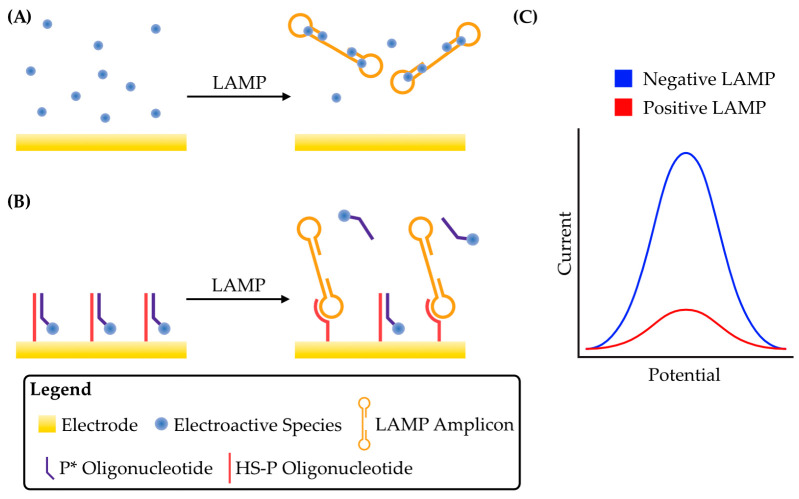
Schematic strategies for the detection of LAMP amplicons by sequestration of an electroactive species from the electrode surface. (**A**) Before LAMP, an electroactive species such as methylene blue or Hoechst 33258 is free to diffuse to the electrode surface, producing a relatively large faradaic current. As LAMP proceeds and amplicons are produced, the electroactive species binds to DNA, reducing its effective concentration and leading to a reduction in peak current. (**B**) An electrode functionalized with an oligonucleotide probe, HS-P. Before LAMP, a complementary oligonucleotide, P*, conjugated to an electroactive species is free to bind HS-P. This concentrates the electroactive species at the electrode surface and gives a large peak faradaic current. As LAMP amplicons are produced, they compete with P* for binding to HS-P and displace them from the surface, reducing the peak current. Adapted from [40]. (**C**) Schematic of a typical voltammogram for both detection strategies. In both cases illustrated in A and B, the net effect is sequestration of the electroactive species from the electrode, and therefore the peak current generated during voltammetry is reduced.

The earliest voltammetric detection of LAMP relied on the principle of end-point detection, i.e., performing the amplification reaction for a set period of time and then detecting its products. Ahmed et al. (2009) exploited an electroactive dye, Hoechst 33258, for the indirect detection of DNA from a genetically modified organism via LAMP [35]. Hoechst 33258 binds AT-rich dsDNA in the minor groove [36], and, at higher concentrations, causes its aggregation [37]. This complexation of Hoechst 33258 with DNA reduces its diffusivity and therefore results in a decrease in the peak faradaic current generated by Hoechst 33258 oxidation during voltammetry that is proportional to the amount of DNA present [38,39]. The first utilization of this phenomenon with LAMP was in an integrated device, whereby the LAMP reaction was first performed in a separate chamber before a valve was opened to mix the reaction contents with Hoechst 33258, and then the entire mixture was subjected to linear sweep voltammetry on a disposable electrochemical printed chip [35]. It was found that LAMP products could be detected after only 20 min of reaction time. It was also shown that the decreased faradaic current for Hoechst 33258 oxidation was entirely due to minor groove binding, suggesting that the larger DNA aggregates previously observed were not required for detection and therefore opened the door for other electroactive compounds that bind dsDNA specifically but do not cause aggregation. The reduced diffusion rate of an electroactive species, and therefore reduced peak faradaic current(s), caused by binding to LAMP amplicons is proportional to the extent of amplification (Figure 2A,C), and this has become a common strategy for voltammetric LAMP detection.

This Hoechst 33258-based assay was adapted to detect meat adulteration in both processed and raw foods, and it was found to be both faster and more sensitive than a PCR-based assay with detection by capillary gel electrophoresis [41]. It has also been extended to the detection of *Vibrio parahaemolyticus*, a foodborne pathogen, in seafood [42]. This device used screen-printed graphene electrodes for their high effective surface area, which increased the sensitivity towards electrochemical redox reactions [43,44]. When tested with DNA extracted from cultured cells and then amplified by LAMP, a detection limit as low as 0.15 CFU/mL was achieved [42]. This device could also detect a single CFU in approximately 80 g of spiked raw seafood after 45 min. The economical design of the electrodes in combination with a portable potentiostat make this a promising device for point-of-use testing.

In other work, microfluidics were integrated with disposable electrochemical printed chips to detect *Escherichia coli* [45]. After extraction from cultured cells by a silica-based kit, the DNA was mixed with LAMP reagents and injected into a microfluidic reaction chamber for amplification. The products were transferred to a detection chamber and mixed with the Hoechst 33258 detection solution for detection by linear sweep voltammetry. This device was able to detect *E. coli* at concentrations as low as 48 CFU/mL after a 35 min reaction time. Additionally, with the idea of urinary tract infection diagnostics in mind, spiked urine samples were also analyzed, and the device achieved a similar limit of detection. When used on two off-target bacterial species, which are species not recognized by the primer set used, a drop in the peak faradaic current was also observed. While it was not as significant as the signal observed for *E. coli* and was attributed to the presence of unamplified genomic DNA binding to Hoechst 33258, this could easily complicate interpretation. More recently, Hoechst 33258 has been used to develop a voltammetric LAMP device for detecting *Mycobacterium tuberculosis* in clinical samples [46]. Purified plasmid DNA or DNA extracted from sputum samples were amplified by LAMP, mixed with Hoechst 33258, and then applied to screen-printed graphene electrodes for detection by cyclic voltammetry. A limit of detection of 40 copies/μL of plasmid DNA was achieved after a 60 min amplification. Furthermore, there was 100% correlation between this LAMP assay and the standard PCR test when analyzing sputum samples. Because the detection is completed with a portable potentiostat and the disposable chips themselves are inexpensive, this is a promising development for point-of-care diagnostics. However, the initial sample processing and requirement for DNA extraction increase complexities that must be overcome.

While end-point detection is perfectly suitable for many applications, the real-time voltammetric detection of LAMP offers some distinct advantages, including both a faster time to detection, since amplification can be confirmed the moment it passes a threshold level, and reduced device complexity, since both the reaction and detection occur in the same chamber without the need to transfer from one to the other. This latter point can also reduce the potential for contamination, depending on device design, since the amplified product does not need to be handled. Hoechst 33258 inhibits DNA replication and is therefore unsuitable for real-time detection. For this reason, other electroactive species have been sought out.

Methylene blue is a dsDNA intercalating dye and was the first electroactive species used in developing a real-time voltammetric LAMP assay [47]. Although the binding mode is different from Hoechst 33258, the principle of voltammetric detection is the same: binding to LAMP amplicons reduces the diffusion rate of the electroactive species and therefore reduces the peak faradaic current(s). In its first use for real-time voltammetric LAMP, the reaction was conducted simply in a 200 μL microfuge tube with a narrow device consisting of screen-printed electrodes inserted into the solution. Amplification above a threshold value could be detected within 25 min by square-wave voltammetry, which was much faster than a comparative voltammetric PCR assay.

Methylene blue intercalation has become the most frequently used strategy for voltammetric LAMP due to its relatively simple mechanism of detection, its applicability for real-time assays, and its commercial availability. For example, this assay could be used to detect as little as two copies of purified *Legionella* spp. genomic DNA, and it could also detect the pathogen in both spiked and environmental water samples [48]. Further focus on the development of integrated devices for simpler point-of-use application led to the combination of a microfluidic LAMP reaction chamber with a three-electrode electrochemical chip [49]. This device, when placed on a heat block, could amplify and detect DNA amplification concurrently through the intercalation of methylene blue, with a detection limit of 4 fg/μL reported for the DNA of the pathogen *Salmonella typhimurium*. Such combination devices have also been integrated with heating elements to create entirely self-contained sensors. A device to monitor pathogenic bacteria in food samples allowed the detection of as little as one copy of *Salmonella enterica* DNA and ten copies of *E. coli* O157 DNA in real food samples [50]. Of note was that detection was unhindered by the turbid food samples, which is a common problem for optical detection systems, and greatly simplifies the required sample preparation. This also has relevance for turbid clinical samples. Further work on detecting pathogens in environmental samples was undertaken in response to the COVID-19 pandemic. Testing wastewater has been a crucial factor in the epidemiological monitoring of viral outbreaks [51]. A device made with screen-printed electrodes was combined with a substrate heater and a portable potentiostat to monitor SARS-CoV2 in wastewater samples [52]. A detection limit of 38 fg/μL for end-point detection and 2.5 fg/μL for real-time detection was achieved. The detection of pathogens in clinical samples has also been an advancement in the development of voltammetric LAMP using methylene blue intercalation. A proof-of-concept device was able to detect as low as 6.18 fg/μL hepatitis B virus DNA extracted from serum samples within 60 min [53]. The device was also validated to detect unextracted DNA by instead using a simple thermal pre-treatment before LAMP to liberate genomic DNA from the viral envelope and nucleocapsid. This can greatly simplify sample processing for point-of-care applications.

Another strategy for displacing an electroactive species from the electrode surface relies not on direct DNA binding, but rather on the interaction between LAMP amplicons and an oligonucleotide conjugated to an electroactive species. The single-stranded loop regions of LAMP amplicons provide an intrinsic advantage over solely dsDNA amplicons found in PCR, for example, since they can readily hybridize with single-stranded oligonucleotide probes [54]. For the construction of such a device, an oligonucleotide complementary to the loop region of a target LAMP amplicon, termed HS-P, was coupled to the surface of an electrode [40]. This oligonucleotide was hybridized with a complementary oligonucleotide (P*) conjugated to methylene blue, termed MB-P*, which brought methylene blue in close proximity to the electrode and therefore generated a high peak faradaic current. In a real-time assay, the generated LAMP amplicons competed with MB-P* for binding to HS-P. As LAMP proceeded and more amplicons were generated, more MB-P* was displaced from the electrode surface and therefore the electrochemical signal decreased (Figure 2B,C). A detection limit of ~3 copies/μL was achieved. The use of another oligonucleotide in addition to the LAMP primers increases the specificity of this device and can reduce the rate of false-positive results. However, this method requires functionalizing of the electrode surface and therefore increases the complexity of sensor preparation.

This strategy, sequestration of an electroactive species from an electrode, provides a simple and straight-forward method for the voltammetric detection of LAMP. The direct binding of an electroactive species to LAMP amplicons eliminates the need to functionalize the electrode, and therefore simplifies device construction. It is adaptable to both end-point and real-time assays, the latter of which is important for faster time-to-detection since a positive single can be detected as soon as it passes a threshold. The detection of less than 10 copies of a template has been reported with high specificity, which can be improved through the use of oligonucleotide probes.

#### 3.1.2. Concentration of Electroactive Species at the Electrode

A strategy related to the sequestration of an electroactive species, although opposite in effect, relies on the concentration of DNA and thus a DNA-binding electroactive species at the electrode surface. In another effort to develop devices for the detection of meat adulteration, ruthenium(III) (RuHex), an electroactive species that binds to DNA electrostatically [55,56], was used in an end-point assay with square-wave voltammetry to give a species-dependent detection limit of 100 to 1 pg/μL [57], which was orders of magnitude more sensitive than a comparable Hoechst 33258 sequestration assay [35,41]. The authors hypothesized that π-stacking interactions between DNA nucleobases and a graphene electrode were responsible for effectively concentrating DNA at the electrode surface [57]. In combination with the DNA-binding RuHex, this in turn led to the concentration of the electroactive species at the electrode surface and a resulting increase in the peak faradaic current. It should be noted, however, that RuHex also coprecipitates with pyrophosphate, a product of LAMP, and this, rather than DNA–graphene interactions, may also be at least partly responsible for the increased peak current observed in this study (discussed in Section 3.2.1) [58].

**Figure 3:**
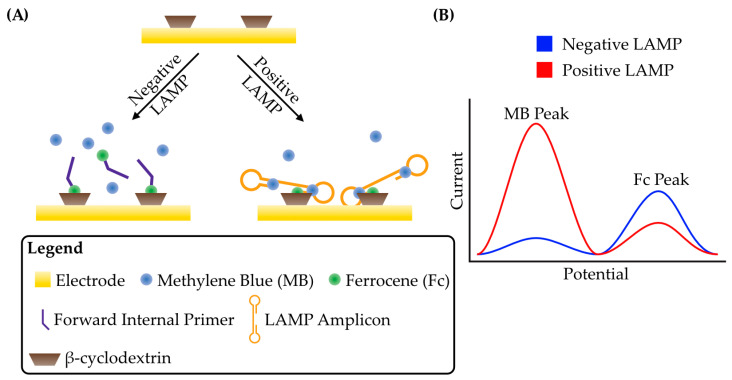
Schematic strategy for the detection of LAMP amplicons by concentrating electroactive species near the electrode surface. (**A**) An electrode is functionalized with β-cyclodextrin. The forward internal primer (FIP) of LAMP is conjugated to ferrocene such that it can be incorporated into the LAMP amplicons. Ferrocene can also bind to β-cyclodextrin. In a negative reaction, Fc-FIP will bind to β-cyclodextrin, producing a relatively large peak faradaic current. Methylene blue in solution will also produce a faradaic current at a different potential. In a positive LAMP reaction, Fc-FIP tethers entire amplicons to the electrode surface. The dsDNA is bound by methylene blue and produces a large ratiometric current between ferrocene and methylene blue. Adapted from [59]. (**B**) Schematic of a typical voltammogram for this detection strategy. Two peaks are observed at different potentials due to the presence of two electroactive species. While ferrocene is brought close to the electrode through interactions with β-cyclodextrin in both positive and negative LAMP reactions, the incorporation of free Fc-labelled primers into LAMP amplicons reduces their diffusivity and therefore the peak faradaic current decreases. For methylene blue, intercalation into LAMP amplicons effectively concentrates it at the electrode surface and results in an increased peak faradaic current.

LAMP amplicons, and, in turn, electroactive species can also be concentrated at the electrode surface by capture either with sequence-specific oligonucleotides or through small molecule interactions. In an implementation of the latter case, a glassy carbon electrode was functionalized with β-cyclodextrin [59], a cyclic oligosaccharide that can form complexes with generally hydrophobic molecules in an aqueous solution, including the electroactive species ferrocene [60]. When ferrocene is conjugated to a LAMP forward internal primer (FIP), it is incorporated into the LAMP amplicons which can then become bound to the electrode surface via interaction with β-cyclodextrin [59]. With the addition of methylene blue, which intercalates between base pairs in the LAMP amplicons, a ratiometric signal of methylene blue and ferrocene (Figure 3A,B) can be measured by square-wave voltammetry that greatly improves reproducibility and gives a detection limit of 0.23 fg/μL for Streptococcus agalactiae genomic DNA. Two independent electrochemical signals can be monitored simultaneously in the ratiometric assay, thus allowing a normalized rather than absolute measurement, and improving linearity between the target DNA concentration and the signal response [61].

This strategy complicates device construction due to the need to functionalize the electrode. It is less commonly used than the sequestration of electroactive species, but the β-cyclodextrin assay described shows an improved limit of detection over many sequestration-based assays. Ratiometric signals should also be possible with oligonucleotide probes, which could provide an additional layer of specificity to the assay.

#### 3.1.3. Comparative Analyses of DNA-Binding Electroactive Species

Several properties of DNA-binding electroactive species are critical for their performance in the detection of DNA amplification [58,62]: (1) a high binding affinity towards dsDNA means more of the electroactive species will be bound, and therefore give a larger signal for a given amount of DNA; (2) for real-time assays, it should not inhibit DNA amplification at the concentration used; (3) it should be chemically and thermally stable in the conditions present during LAMP extension; (4) it should have a stable electrochemical signal over the duration of LAMP; and (5) it should have a redox potential within the accessible range of the aqueous solution. Although many of these properties can be determined independently, the utility of a specific DNA-binding electroactive species for LAMP may often be a compromise between them, and the performance of an electroactive species is therefore best evaluated in conjunction with LAMP itself.

Many electroactive compounds with DNA-binding properties have been employed for the voltammetric detection of LAMP; however, because of the widely varying experimental conditions of individual studies (target DNA, primer sets, detection method, etc.), it is difficult to assess them comparatively across individual studies. Fortunately, some systematic, comparative studies have been conducted to evaluate the relative merits of some of these electroactive species when used for voltammetric LAMP detection. A study of 10 electroactive species found that those which bind dsDNA through intercalation between the nucleobases tended to have a higher sensitivity and faster threshold times than the non-intercalating electroactive species studied [58]. Additionally, higher binding affinity, as quantified by the equilibrium association constant (K_A_), generally correlated with faster threshold times since more of the electroactive species was bound by the comparatively smaller amount of DNA present earlier in the time course of LAMP. However, there were diminishing returns when binding affinity was too high due to an inhibitory effect on amplification efficiency that resulted in lower sensitivities and lower threshold times. A similar, affinity-dependent effect has also been observed with fluorescent dyes in both LAMP [63] and PCR [64]. Tighter binding fluorescent dyes increased the melting temperature of the DNA duplex, indicating a stabilizing effect, and it was hypothesized that they may therefore have an affect on primer hybridization or polymerase activity. Amongst three intercalating electroactive species, including the osmium complex [Os(bpy)_2_dppz]^2+^ (K_A_ ~ 5 × 10^6^ M^−1^ [62]), the methylene blue derivative PhP (K_A_ ~ 5 × 10^5^ M^−1^ [65]), and methylene blue (K_A_ ~ 2 × 10^4^ M^−1^ [66]), all were found to have similar average threshold times despite ranging in affinity over two orders of magnitude. This was due to the LAMP inhibition that was correlated with DNA-binding affinity [58]. In fact, the inhibitory effect of [Os(bpy)_2_dppz]^2+^ was quite pronounced even at low concentrations, and high inhibition has also been observed for the analogous ruthenium complex [Ru(bpy)_2_dppz]^2+^ [67]. In subsequent work, both [Os(bpy)_2_dppz]^2+^ and PhP were capable of detecting single copies of target DNA in a voltammetric LAMP assay, but the inhibitory effect of the former along with signal instability made PhP more desirable for high-sensitivity detection [68]. Methylene blue also showed high-sensitivity detection and the lowest degree of inhibition. Coupled with its wide commercial availability, methylene blue has remained an attractive choice for voltammetric LAMP.

Purpose-designed electroactive species have also been synthesized for voltammetric LAMP with the intention of fine-tuning affinity while avoiding the inhibitory properties of species such as [Os(bpy)_2_dppz]^2+^ [69]. Naphthoquinone-imidazole was dimerized via different linkers to generate bis-intercalating electroactive species: electroactive molecules with two DNA intercalating moieties that increase binding affinity. These bis-intercalating molecules had affinities ranging from K_A_ = 5.5 × 10^4^ to 6.0 × 10^4^ M^−1^ which were approximately 3- to 7-fold higher than their mono-intercalating counterparts. They were also thermostable at the LAMP extension temperature of 65 °C and not inhibitory up to at least 20 μM. While both methylene blue and the bis-intercalating electroactive species achieved a detection limit of one copy/μL of *S. enterica* DNA, these new probes had threshold times for detection less than half that of methylene blue, 10 vs. 22 min, which can be a significant advantage for point-of-use applications.

The selection of electroactive species can have a large impact on assay performance. Although [Os(bpy)_2_dppz]^2+^ and PhP showed lower limits of detection than methylene blue in comparative assays, the difference was not extreme, and the wide availability of methylene blue makes it a popular choice. It also showed the lowest degree of LAMP inhibition due to its lower binding affinity. Bespoke, bis-intercalating electroactive species have been shown to improve assay time significantly, but their preparation needs to become more accessible before they can be readily adopted.

### 3.2. Monitoring non-DNA LAMP Products

#### 3.2.1. Pyrophosphate Detection

The electroactive species ruthenium(III) (RuHex) was used quite early in the development of voltammetric LAMP [70]. Square-wave voltammetry was conducted concurrent with LAMP amplification, allowing as low as 30 copies/μL of *E. coli* genomic DNA and 20 copies/μL of *Staphylococcus aureus* genomic DNA to be detected within 30 min in real time. The positively charged RuHex binded DNA electrostatically via the negatively charged phosphodiester backbone with affinity K_A_ = 6.0 × 10^4^ to 1.2 × 10^6^ M^−1^ over Na^+^ concentrations from 100 to 0 mM [56]. The mechanism was initially thought to be similar to the first-used Hoechst 33258 or the now more commonly used methylene blue assays: sequestration of RuHex by LAMP amplicons reduced their diffusivity and therefore reduced the peak faradaic current [70]. However, it was later found that the electrochemical signal generated by RuHex during LAMP was the result of two distinct processes: an initial reduction in the peak faradaic current due to DNA binding early in amplification, as expected, followed by a much larger reduction in the current later in amplification [58]. This latter reduction was determined to be from the complexation and coprecipitation of RuHex with pyrophosphate: a product of nucleoside triphosphate in addition to the nascent chain during DNA replication.

**Figure 4:**
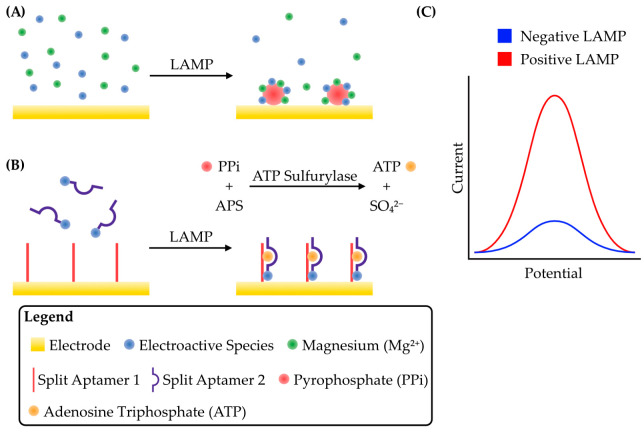
Schematic strategies for the detection of pyrophosphate produced through DNA replication during LAMP. (**A**) Before LAMP, the electroactive species RuHex diffuses freely. Mg^2+^, a cofactor for DNA polymerases, is also present. As LAMP proceeds, RuHex coprecipitates onto the electrode surface with Mg^2+^ and the pyrophosphate produced from DNA polymerization, concentrating RuHex on the electrode and greatly increasing the peak faradaic current. Adapted from [72]. (**B**) An ATP-binding aptamer is split in two, with one half (Split Aptamer 1) conjugated to the electrode while the other (Split Aptamer 2), conjugated to an electroactive species, is free in solution. The enzyme ATP sulfurylase catalyzes a reaction between the pyrophosphate (PPi) produced during LAMP and adenosine phosphosulfate (APS) to produce ATP and sulfate (SO_4_^2−^). The ATP brings the split aptamer together, concentrating the electroactive species on the electrode and producing a large peak current. Adapted from [75]. (**C**) Schematic of a typical voltammogram for these detection strategies. Both strategies result in a concentration of an electroactive species at the electrode surface, either by coprecipitation in A or by aptamer formation in B. The net result in both cases is an increase in peak faradaic current during voltammetry.

Compared with intercalating probes such as methylene blue, RuHex was found to be less sensitive for voltammetric LAMP since its binding affinity to DNA was relatively weak at higher salt concentrations and the relatively high concentration of pyrophosphate required for detection occurred much later in the amplification process [68]. The relatively low affinity of RuHex for either DNA or pyrophosphate means that higher concentrations of this electroactive species are necessary for sensitive LAMP detection. It has been observed, however, that concentrations higher than 60 μM were inhibitory to amplification by the *Bst* polymerase commonly used for LAMP [70]. A different strand-displacing polymerase, GspSSD, was found to be more tolerant of DNA-interacting species, and concentrations as high as 1 mM RuHex could be used without meaningful polymerase inhibition, thus allowing a more stable and sensitive assay [71]. It was also observed that the binding mode of RuHex (binding DNA vs. coprecipitation with pyrophosphate) was dependent on magnesium cation concentration. At lower concentrations, RuHex preferably bound LAMP amplicons leading a sequestration effect that reduced the peak faradaic current; however, at higher concentrations, RuHex coprecipitated with pyrophosphate and magnesium ions on the electrode surface, leading to increased peak faradaic currents (Figure 4A,C). When used with linear sweep voltammetry, the repeatable detection of as low as 2 copies/μL could be achieved.

This detection strategy was used to create a novel multiplex device for detecting multiple target miRNAs [72]. A device with multiple, independent sets of working, reference, and counter electrodes was used. Five distinct primer sets were spotted and adsorbed onto the surface of the device within a liquid flow channel. Injecting a complex mixture of miRNAs and LAMP reagents through the channel exposed all five primer spots to the components necessary for amplification, but, importantly, limited migration of the primer sets within the channel and thus created five spatially distinct reactions that could be monitored independently [72,73]. Amplification was completed by *Tin* strand-displacing DNA polymerase with 1 mM RuHex, which coprecipitated on the electrode surfaces with pyrophosphate and magnesium and allowed detection of 10^3^ to 10^6^ copies of target miRNAs. A similar device was used to monitor the expression levels of specific miRNAs in cancer patients [74].

Aside from RuHex precipitation, another strategy for pyrophosphate detection involves precipitation of inorganic phosphate with acidic molybdate in an end-point assay [76]. A thermostable pyrophosphatase was included in the LAMP reaction so that pyrophosphate produced during DNA replication was hydrolyzed to phosphate. When the LAMP products were mixed with molybdate, a precipitate formed on the surface of a carbon nanotube-functionalized glassy carbon electrode which could be detected by cyclic voltammetry in a standard electrochemical cell. This strategy allowed the detection of *Nosema bombycis* genomic DNA, a pathogen relevant to sericulture, with a detection limit of 17 fg/μL. The sensitivity of *N. bombycis* DNA detection was improved with a so-called split aptamer-based electrochemical sandwich assay, which is also based on pyrophosphate detection [75]. Briefly, an ATP-binding aptamer was split in two, with one section attached to an electrode and the other to a methylene blue-functionalized gold nanoparticle. In the presence of ATP, the complete aptamer reformed and thus concentrated methylene blue at the electrode surface, thus giving a voltammetric signal. For this assay to detect LAMP, the pyrophosphate produced was transferred to adenosine 5′-phosphosulfate by ATP sulfurylase, thus generating ATP and sulfate (Figure 4B,C). The ATP concentration is therefore directly proportional to the extent of DNA amplification. The detection limit was improved ~36-fold over the molybdate assay to 0.47 fg/μL.

The detection of pyrophosphate by RuHex coprecipitation has been developed into a sensitive assay with detection limits as low as 2 copies/μL of template. The multiplex assay developed with this methodology allows it to detect multiple targets in a complex sample and should also be adaptable for use with other strategies such as sequestration of an electroactive species by direct DNA binding. The novel split-aptamer strategy shows an improved limit of detection over precipitation strategies, but also complicates device construction and could have implications on storage longevity depending on enzyme stability.

#### 3.2.2. Proton (pH) Detection

Since a product of DNA replication is protons, the amplification of target DNA results in a decrease in pH in weakly buffered reaction mixtures. Although potentiometric measurements are standard for detecting pH changes, creative voltammetric methods have also been designed to be pH-responsive. One recently created device relies on the pH-dependent oxidation of the electroactive species phenol red [77]. Phenol red is commonly used as a colorimetric pH indicator for monitoring LAMP, either by naked-eye observation or by more quantitative absorbance measurements. It is also, however, electroactive, and the peak potentials of its reduction and oxidation reactions are pH-dependent. The determination of the peak oxidation potential of end-point LAMP reactions containing phenol red by cyclic and linear sweep voltammetry on screen-printed electrodes was used to monitor DNA amplification. Used to amplify *Streptococcus pneumoniae* DNA, this assay achieved a limit of detection of 2 × 10^5^ copies/μL for both purified plasmid and spiked urine samples. Although this method used end-point detection, it should be adaptable to real-time monitoring of the LAMP reaction for a more rapid assay.

**Figure 5:**
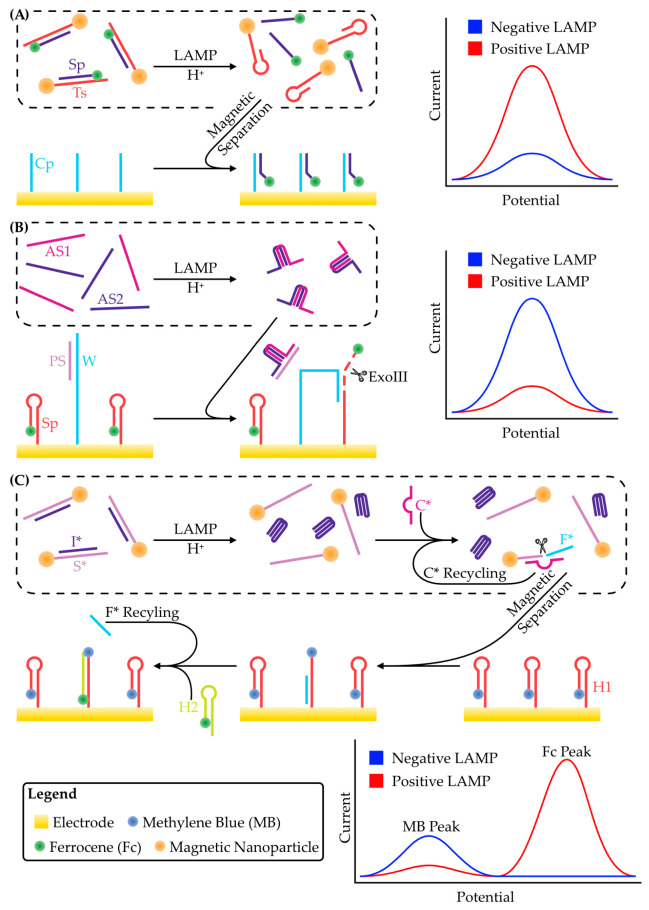
Schematic strategies for the detection of protons produced through DNA replication during LAMP. (**A**) An oligonucleotide, Ts, is conjugated to a magnetic nanoparticle. Another oligonucleotide, Sp, is conjugated to ferrocene and hybridized to Sp. This duplex is pH sensitive, and protons produced during LAMP cause it to denature. Ts is removed by magnetic separation and the purified Sp is hybridized to another oligonucleotide, Cp, that is functionalized to the electrode. This concentrates the Sp-bound ferrocene at the electrode surface and produces a large peak faradaic current, as shown in the schematic voltammogram. Adapted from [78]. (**B**) The electrode is functionalized with two oligonucleotides: W and Sp. Sp is conjugated to ferrocene and forms a hairpin structure, bringing ferrocene in close contact with the electrode. W is blocked by another oligonucleotide, Ps. Protons produced during LAMP cause two oligonucleotides (AS1 and AS2) to form an i-motif. This i-motif has a region complementarity with Ps and causes its release from W via competitive binding. W can then interact with Sp, breaking the latter’s hairpin structure and making it susceptible to exonuclease III digestion. This releases ferrocene from the electrode and results in a decrease in peak current, as shown in the schematic voltammogram. W is then free to interact with more Sp, releasing more ferrocene, and amplifying the signal through a walking mechanism. Adapted from [80]. (**C**) An oligonucleotide, S*, is conjugated to a magnetic nanoparticle. Hybridized to S* is another oligonucleotide, I*. Acidification of the solution following LAMP causes I* to form an intramolecular i-motif which favours its release from S*. This allows another oligonucleotide, C*, to hybridize to S*. Together, this duplex forms an autocatalytic deoxyribozyme that releases a short segment of S*, called F*. F* is purified by magnetic separation of S*. An electrode has been functionalized with a hairpin forming oligonucleotide, H1, that is conjugated to methylene blue. The purified F* is applied to the electrode where it can hybridize with H1, breaking the hairpin and displacing methylene blue from the electrode surface. This reduces the peak faradaic current as shown in the schematic voltammogram. Breaking the H1 hairpin also enables another oligonucleotide, H2, which is conjugated to ferrocene, to displace F* and hybridize with H1. This brings ferrocene in close contact with the electrode, increasing its peak faradaic current. The released F* is then free to break more H1 hairpins, amplifying the signal. Adapted from [81].

Other strategies for pH-dependent voltammetric LAMP exploit the pH-sensitivity of nucleic acid hybridization. Two complementary oligonucleotides were constructed, with one, termed Ts, conjugated to a magnetic nanoparticle and the other, termed Fc-SP, to the electroactive species ferrocene [78]. At pH 8, these oligonucleotides formed a stable hybrid, but when exposed to a positive LAMP reaction, the decreased pH caused denaturation of the complex. Ts can be removed by magnetic separation, and the liberated Fc-SP was hybridized to the working electrode of an electrochemical biosensor that had been functionalized with another complementary oligonucleotide, Cp (Figure 5A). With square-wave voltammetry, a limit of detection of 0.31 fg/µL for *N. bombycis* DNA was achieved, which was much more sensitive than a comparable assay using a simple potentiometric pH metre [79].

Similar assays have been developed utilizing the i-motif: a quadraplex DNA structure that forms at acidic pH [82]. In this structure, hemiprotonated cytosine base pairs can form and allow the hybridization of two polycytosine strands, which can then intercalate with another hemiprotonated cytosine duplex to form the quadraplex. As protonation is required for this type of base pairing, the structure is pH-sensitive and can therefore be used to monitor LAMP. For voltammetric detection, i-motif formation must result in a change of the rate of oxidation or reduction of some electroactive species. To create a biosensor based on this phenomenon, two oligonucleotides were coupled to the surface of a gold-coated glass carbon electrode [80]. The first, termed Fc-SP, was conjugated to electroactive ferrocene and formed a hairpin structure that concentrated ferrocene at the electrode surface and generated a strong peak faradaic current. The second, termed W, had a region of complementarity with Fc-SP, but hybridization was prevented by the presence of a third oligonucleotide, termed PS. The formation of the i-motif from two separate DNA oligonucleotides due to protons released during LAMP created a region of complementarity to the PS strand that caused its displacement from W. The Fc-SP complementary region of W was then free to interact with Fc-SP, which broke the latter’s hairpin structure and made it susceptible to exonuclease III digestion. This, in turn, released ferrocene from the electrode with a concomitant decrease in the peak faradaic current. It also freed W to interact with another Fc-SP oligonucleotide, amplifying the signal via a so-called walking mechanism [83,84] whereby each PS-liberated W strand can move sequentially from one Fc-SP to the next, releasing ferrocene along the way (Figure 5B). The device achieved a detection limit of 0.018 fg/μL [80].

Another i-motif-based strategy involves the self-assembly of a deoxyribozyme in response to protons released by LAMP [81]. A magnetic nanoparticle was functionalized with an oligonucleotide, termed S*, which was hybridized with another, cytosine-rich, oligonucleotide, I*. Acidification by LAMP allowed the formation of i-motifs by I* which favoured its dissociation from S*. This freed S* to bind another complementary oligonucleotide, C*. The S*:C* hybrid was an autocatalytic deoxyribozyme [85], and, in the presence of magnesium cations, cleaved S* to release both C*, which was then free to bind another S*, and a short fragment of S*, termed F* [81]. F* was then purified by magnetic separation and applied to an electrode functionalized with a methylene blue-conjugated hairpin oligonucleotide, MB-H1. The binding of F* to MB-H1 broke the hairpin and moved methylene blue away from the electrode surface, resulting in a decreased peak faradaic current as measured by square-wave voltammetry. The disruption of the MB-H1 hairpin also allowed another free-floating oligonucleotide conjugated to ferrocene, Fc-H2, to hybridize with MB-H1 and bring ferrocene in close contact with the electrode, increasing the peak faradaic current (Figure 5C). Thus, a ratiometric signal could be measured that minimized background noise and gave a limit of detection of 0.013 fg/μL. While these pH-sensitive hybridization-based assays have achieved incredible sensitivity, their multistep methodology and the requirement to functionalize the electrode increase their complexity and time required for testing.

The strategies to monitor DNA amplification by the pH sensitivity of oligonucleotide hybridization have achieved some of the lowest limits of detection for voltammetric LAMP assays. The methods exploiting the i-motif in particular, which include a signal amplification outside of the exponential DNA replication of LAMP, have very low limits of detection. However, their complex construction and multistep procedure may limit their suitability in point-of-use applications where turnaround time and ease-of-use are important factors.

## 4. Conclusions and Future Directions

Much work has been done in combining LAMP with voltammetric electrochemical detection. Exploiting the advantages of both LAMP, especially its rapid, high-efficiency amplification, and voltammetry, including high sensitivity and the ability to be measured by cheap and disposable devices, has allowed the creation of biosensors that can detect pathogenic organisms with high selectivity and detection limits that rival standard fluorescent assays for both LAMP and PCR, all of which have been reported to achieve single-copy detection. Because sensitivity and specificity depend in large part on the primer design and concentrations, the choice of polymerase, and magnesium concentration, systematic studies of all three methods will be necessary for accurate comparison. Threshold times for voltammetric LAMP are also comparable to fluorescent assays, and the total turnaround time is more affected by steps such as sample preparation and handling than the amplification time itself. The monitoring of LAMP by direct DNA-binding electroactive species is by far the most common detection strategy and can achieve real-time, rapid detection with high sensitivity, and its conceptual simplicity makes the development of new devices accessible. In terms of methodology, it is comparable to fluorescent LAMP assays in which an intercalating dye or other fluorescent probe is added directly to the reaction mixture and no further processing steps are required for detection. However, voltammetric LAMP detection has also proven adaptable to more creative assays involving complex DNA structures that could provide even greater sensitivity at the expense of time-to-detection.

A drawback of many existing voltammetric LAMP assays is the laborious sample preparation conducted before amplification and detection can take place. In contrast to other point-of-use diagnostic tests such as LFAs, for example, which can be performed by untrained individuals and achieve detection within minutes, LAMP assays can often involve extraction and purification of the genomic DNA or RNA from the pathogen before detection can occur. The standard methods for this require training and laboratory equipment and can therefore preclude its applicability to many point-of-use applications. However, because of LAMP’s relatively high tolerance for contaminants, simplified extractive procedures are feasible [86,87]. Some existing studies with voltammetric LAMP have applied these principles, for example, using heat pre-treatment to release DNA from hepatitis B virions in positive serum samples [53] or from *Salmonella* spp. in contaminated food samples [50] without the need for a separate extraction step. Additionally, because there is some evidence that LAMP conducted without nucleic acid extraction can result in lower sensitivity [88], recent work with voltammetric LAMP has, instead of eliminating RNA extraction and purification, sought to simplify it by utilizing magnetic bead-based purification that minimizes the equipment required and decreases turnaround time [89]. For ease and speed-of-use, further work towards point-of-use devices should focus on integrating simplified extraction protocols with voltammetric LAMP, and detection strategies such as the direct DNA binding of electroactive species may additionally be preferable over more complicated, multistep assays for this specific use case.

Despite LAMP’s theoretically high specificity, reports of false-positive results occur [33]. Carryover contamination can be problematic, as can contamination during sample preparation, and/or manipulation during measurements [86]. Integrated and disposable devices with as little sample handling as possible can serve to mitigate this problem. However, there may also be intrinsic difficulties with LAMP itself. The requirement for at least four relatively long primers increases the likelihood of primer dimers and self-priming intramolecular hairpin structures which can be amplified and therefore detected [90]. As these are amplified with less efficiency than actual target DNA, time-gating in real-time LAMP has been proposed to accept as positive only those samples in which amplification is detected within a set time period [86,91]. Additionally, DNA melting curves can distinguish real amplicons from amplified primers [91], and they are easily collected after voltammetric LAMP with the same electroactive probes and on the same device [59,68]. LAMP could be combined with other assays for improved specificity [17], and it has also been shown that using multiple primer sets in a single LAMP reaction can improve both specificity and time-to-detection [92]. Methods that rely on sequence-specific DNA probes, such as the voltammetric detection reported by Liu et al. (2020) (Figure 2B) [40], should also provide an additional layer of specificity.

Finally, while voltammetry is a routine procedure in an electrochemical laboratory using a stationary potentiostat, adapting it to point-of-use applications for LAMP-based assays is an important avenue for further research. A handful of commercial, voltammetric LAMP-based nucleic acid analyzers are already available; however, again, the scale and cost of the equipment can be more suited to a laboratory setting [54]. Electrochemical biosensors lend themselves towards miniaturization, and, in combination with portable, handheld potentiostats, voltammetric LAMP can also be adapted for use outside of the laboratory [93]. As reviewed here, much work has been done with proof-of-concept devices for detecting pathogens in a point-of-use format, but more development to integrate the various steps, from sample handling to detection, and more evaluation in clinical settings need to be completed before they can be brought into widespread use. Nevertheless, voltammetric LAMP, and electrochemical LAMP more generally, remain a promising avenue of research in developing inexpensive point-of-use devices that combine sensitivity, rapidity, and simplicity.

## Data Availability

Not applicable.

## Abbreviations

ATP: adenosine triphosphate, APS: adenosine phosphosulfate, CFU: colony-forming unit, dsDNA: double-stranded DNA, Fc: ferrocene, FIP: forward internal primer, K_A_: equilibrium association constant, LAMP: loop-mediated isothermal amplification, LFA: lateral flow assay, MB: methylene blue, miRNA: micro RNA, PCR: polymerase-chain reaction, PPi: pyrophosphate, QCM: quartz crystal microbalance, RuHex: hexaammine ruthenium(III).
